# Recovery of Steroidal Alkaloids from Potato Peels Using Pressurized Liquid Extraction

**DOI:** 10.3390/molecules20058560

**Published:** 2015-05-13

**Authors:** Mohammad B. Hossain, Ashish Rawson, Ingrid Aguiló-Aguayo, Nigel P. Brunton, Dilip K. Rai

**Affiliations:** 1 Department of Food Biosciences, Teagasc Food Research Centre, Ashtown, Dublin D15, Ireland; E-Mails: mohammad.hossain@teagasc.ie (M.B.H.); ashishrawson@gmail.com (A.R.); ingrid.aguilo@irta.cat (I.A.-A.); 2 Indian Institute of Crop Processing Technology, Thanjavur 613005, TN, India; 3 IRTA, XaRTA-Postharvest, Edifici Fruitcentre, Parc Científic i Tecnològic Agroalimentari de Lleida, Lleida 25003, Catalonia, Spain; 4 School of Agriculture and Food Science, University College Dublin, Dublin D4, Ireland

**Keywords:** glycoalkoids, potato peels, pressurized liquid extraction, response surface methodology

## Abstract

A higher yield of glycoalkaloids was recovered from potato peels using pressurized liquid extraction (1.92 mg/g dried potato peels) compared to conventional solid–liquid extraction (0.981 mg/g dried potato peels). Response surface methodology deduced the optimal temperature and extracting solvent (methanol) for the pressurized liquid extraction (PLE) of glycoalkaloids as 80 °C in 89% methanol. Using these two optimum PLE conditions, levels of individual steroidal alkaloids obtained were of 597, 873, 374 and 75 µg/g dried potato peel for α-solanine, α-chaconine, solanidine and demissidine respectively. Corresponding values for solid liquid extraction were 59%, 46%, 40% and 52% lower for α-solanine, α-chaconine, solanidine and demissidine respectively.

## 1. Introduction

**Figure 1:**
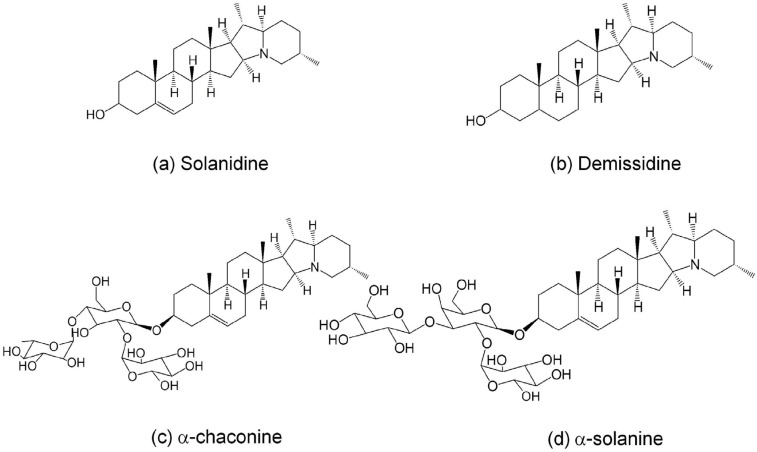
Chemical structures of glycolkaloids in potato peels.

Potato constitutes an integral part of diets in many countries around the world. Potatoes are rich in carbohydrate and contain high quality proteins as well as antioxidative polyphenols, vitamins and minerals [1]. Whilst potatoes are nutritionally rich food source they also possess a group of toxic compounds called steroidal alkaloids which are found predominantly in the skin [2]. Steroidal alkaloids are secondary metabolites found mainly in the plants of *Solanaceae* family of which α-solanine and α-chaconine are the most abundant in potato tubers (Figure 1). In the plant they function as defence molecules against bacterial [3], fungal [4] and insect attacks [5]. However, in humans toxic symptoms can include colic pain in the abdomen and stomach, gastroenteritis, diarrhoea, vomiting, fever, rapid pulse, low blood pressure to neurological disorders [6,7]. Notwithstanding their toxicity following ingestion studies, in the last decade have demonstrated that these compounds may also possess beneficial properties such as anticancer [8] and anti-inflammatory effects [9] depending on dose and conditions of use [2]. Therefore, maintaining or even enhancing these bioactivities whilst minimizing toxicity by synthetic modifications of the steroidal alkaloids could be crucial for their applications in the phytopharmaceutical industries. Food processing industries particularly potato-based snacks manufacturing industries generate a huge volume of potato peel as by-product. In fact it is estimated between 70 and 140 thousand tonnes of peels are generated worldwide annually [10]. In general the current practice is that the potato peel by-products are discarded in land-fills with accompanying environmental consequences or used as low value animal feed [1]. In common with other high volume uniform plant by-products, food processors, government agencies and downstream processors capable of recovering value from by-products are becoming aware of the potential of potato peel as a repository for chemicals with commercial value. Utilization of potato peels for the extraction of steroidal alkaloids will reduce the disposal problem while paving the way for new ingredients for use in the phytopharmaceutical industry.

Similar to other phytochemicals, traditionally steroidal alkaloids are extracted using conventional solid-liquid extraction (SLE) at atmospheric pressures [11,12]. SLE of phytochemicals from plant matrices is influenced by several factors including the extraction solvent, temperature, time, pressure, particle size and solvent to sample ratio [13]. The SLE technique uses high volumes of often toxic solvents and is time consuming and requires significant manual input. Pressurized liquid extraction (PLE) uses low-boiling solvents or solvent mixtures at elevated temperatures of up to 200 °C and pressure (3000 psi) to extract target compounds. The high pressure or temperature or their combination thereof increases target compound solubility, solvent diffusion rate and mass transfer, while solvent viscosity and surface tension decrease. In many cases PLE presents some advantages over conventional SLE such as usage of lower quantities of toxic organic solvents requiring shorter extraction times with higher selectivity and/or extraction yields. In addition PLE is automated and retains samples in an oxygen- and light-free environment [14]. From an industrial perspective however, to date, sufficiently up-scaled versions of the PLE equipment for large scale recovery of compounds have not been available. However, with the rapid advancement of science and technology, large scale PLE instrumentation is possible in the future. A considerable number of studies have also focussed on the use of PLE from an environmental perspective [15,16,17]. In fact several recent studies have reported the use of PLE to extract antioxidant phenolic compounds from potato peels [18,19,20]. However, none have concentrated on maximising the extraction of steroidal alkaloids from potato peel using PLE. To maximise recovery of a particular target compound key external PLE extraction conditions such as temperature and solvent composition must be optimised. Response surface methodology (RSM) is a statistical technique which allows the user to identify optimal conditions for a selected response while minimizing the number of experiments required. In the present study, optimisation of solvent concentration and temperature for extracting steroidal alkaloids from potato peels was carried out using pressurized liquid extraction assisted by response surface methodology.

## 2. Results and Discussion

### 2.1. Extraction of Steroidal Alkaloids from Potato Peels Using Pressurized Liquid

Among the four common steroidal alkaloids in potato peel (Figure 1), α-chaconine was the most abundant followed closely by α-solanine. The alkaloidal aglycone (solanidine) and its dihydrogenated form (demissidine) were present in relatively low concentrations in the extracts analysed. In fact, the content of α-chaconine was approximately 10 times higher than that of demissidine. It is however debatable whether the steroidal glycoalkaloids (α-chaconine and α-solanine) or the aglycone alkaloids are biologically more important. Recent findings suggested that the aglycone alkaloids have higher anti-inflammatory activity than their glycosylated counterparts [12]. On the other hand, the glycoalkaloids are more potent in their anticarcinogenic activity [2].

As many as 11 different solvents of varying polarity were assessed for their efficacy on the extraction of glycoalkaloids from potato peels. The results showed that the methanol was the best extractant whilst acetonitrile and chloroform were the poorest extractant for glycoalkaloids (Table 1).

**Table 1:** Efficacy of various solvents in the extraction of glycoalkaloids from potato peels.

Extracts Obtained by 1 g Dried Potato Peel (DPP) in 25 mL for 24 h	α-Solanine	α-Chaconine	Solanidine	Demissidine	Total	Extraction Yield (mg/g DPP)	Total Glycoalkaloid (µg/g DPP)
	(µg/mg Dried Extract)						
Tetrahydrofuran	0.26 ± 0.01	2.55 ± 0.18	0.97 ± 0.10	0.14 ± 0.01	3.91 ± 0.23	47.20 ± 0.75	184.55 ± 3.41
Methanol	4.29 ± 0.21	3.28 ± 0.26	2.40 ± 0.26	0.15 ± 0.02	10.12 ± 0.45	75.20 ± 2.10	761.02 ± 5.87
Ethylacetate	0.14 ± 0.01	0.03 ± 0.001	1.40 ± 0.21	0.14 ± 0.03	1.70 ± 0.14	38.40 ± 1.95	65.28 ± 1.62
Chloroform	0.14 ± 0.01	0.03 ± 0.002	1.86 ± 0.16	0.15 ± 0.02	2.18 ± 0.26	23.20 ± 1.65	50.58 ± 2.41
Acetonitrile	0.15 ± 0.02	0.04 ± 0.001	1.17 ± 0.09	0.14 ± 0.01	1.47 ± 0.19	34.40 ± 2.71	50.57 ± 2.46
Acetone	0.17 ± 0.02	2.30 ± 0.21	1.72 ± 0.07	0.15 ± 0.03	4.34 ± 0.35	31.20 ± 3.10	135.41 ± 3.55
Iso-propanol	1.01 ± 0.15	1.86 ± 0.19	0.94 ± 0.10	0.13 ± 0.01	3.94 ± 0.30	49.60 ± 2.95	195.42 ± 4.10
Ethanol	4.06 ± 0.31	3.90 ± 0.11	1.49 ± 0.22	0.14 ± 0.02	9.59 ± 0.42	41.60 ± 3.87	398.94 ± 3.86
Dichloromethane	0.86 ± 0.1	0.31 ± 0.02	0.68 ± 0.01	0.13 ± 0.01	1.97 ± 0.11	40.80 ± 2.52	80.38 ± 2.71
Water	0.69 ± 0.05	0.03 ± 0.001	0.54 ± 0.02	0.12 ± 0.01	1.38 ± 0.20	101.60 ± 5.20	140.21 ± 1.96
5% Acetic acid	2.98 ± 0.22	1.54 ± 0.15	0.69 ± 0.01	0.12 ± 0.01	5.33 ± 0.31	138.40 ± 5.92	737.67 ± 6.84

**Figure 2:**
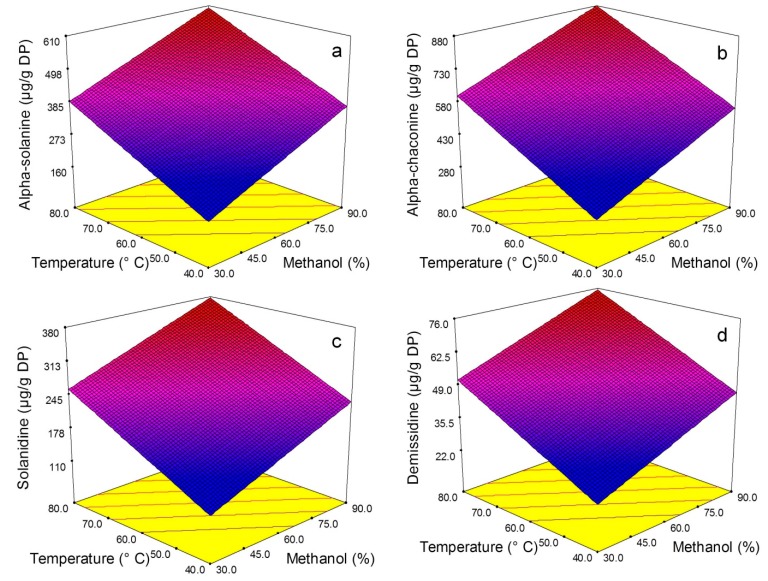
Response surface plots of potato peel pressurized liquid extracts showing the effect of temperature and methanol concentration on α-solanine (**a**), α-chaconine (**b**), solanidine (**c**), and demissidine (**d**) contents.

Figure 2a–d presents response surface plots showing the effect of temperature and methanol concentration (percentage of methanol in water) on the content of four steroidal alkaloids: α-solanine, α-chaconine, solanidine and demissidine in PLE extracts of freeze dried potato peel. As illustrated in Figure 2 increasing temperature and the proportion of methanol resulted in increased recovery of all four alkaloidal compounds. However, there was no interaction between the factors on the parameters studied. The pattern of effect was best fitted with a linear model for all four alkaloids.

**Figure 3:**
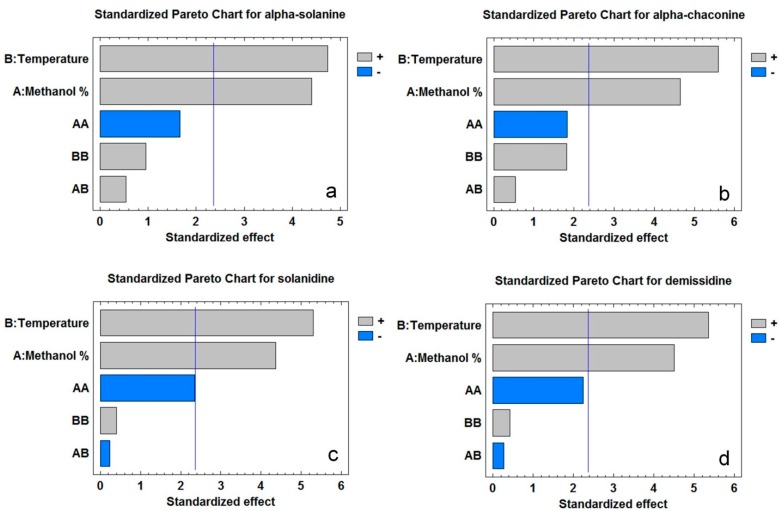
Standardised pareto charts of potato peel showing the effect of different factor terms (temperature and methanol concentration) on steroidal alkaloids such as α-solanine (**a**), α-chaconine (**b**), solanidine (**c**), and demissidine (**d**) contents in pressurized liquid extracts. Bars exceeding the vertical line on the graph indicate that the corresponding factor terms are significant (*p* < 0.05).

Figure 3a–d presents standardised pareto charts for the two factors (% methanol in water and temperature) investigated for their effect on the individual steroidal alkaloids content in the PLE extracts. Temperature had a greater effect than methanol concentration on alkaloid recovery in all cases (Figure 3a–d). In fact, when temperature was increased from 40 °C to 80 °C (the highest temperature used in the current study) keeping the methanol concentration constant at 60%, levels of individual alkaloids increased by 70.95% to 87.07%. The total steroidal alkaloid content of the extract was increased by 78.68%. Extraction yield was also up by 77.31% with this increase in temperature. The total steroidal alkaloid contents of PLE and SLE of the current study were in the range (585 to 5342 µg/g dried peel (DP)) as reported by Deußer *et al.* [21] in the peels of 16 potato varieties grown in Luxemburg. The huge variability of the steroidal alkaloids among potato samples due to differences in growing conditions, postharvest storage and varieties makes their comparison unrealistic. So far there has been no study on the effect of temperature on the PLE of steroidal alkaloids from potato peel. However, several studies using PLE reported the significant increases of antioxidant phenolics in the extracts from plant materials as a result of enhanced temperatures [20,22,23]. This is not surprising since increasing temperatures and thereby enhance the solubility of many compounds. Temperature also disrupts analyte-sample matrix interaction caused by van der Waals forces, hydrogen bonding and dipole attraction [24]. This facilitates bound analytes release from the sample matrix to the extraction solvent enhancing extraction efficiency. High temperatures might also have increased the diffusion rate of the compounds resulting in compounds being extracted at a higher rate. Using high pressure in PLE might increase extraction by disrupting the matrix allowing mass transfer of the analyte from the sample into the solvent. High pressure also minimizes the formation of air bubbles which hinder the solvent to reach the analyte [18].

**Figure 4:**
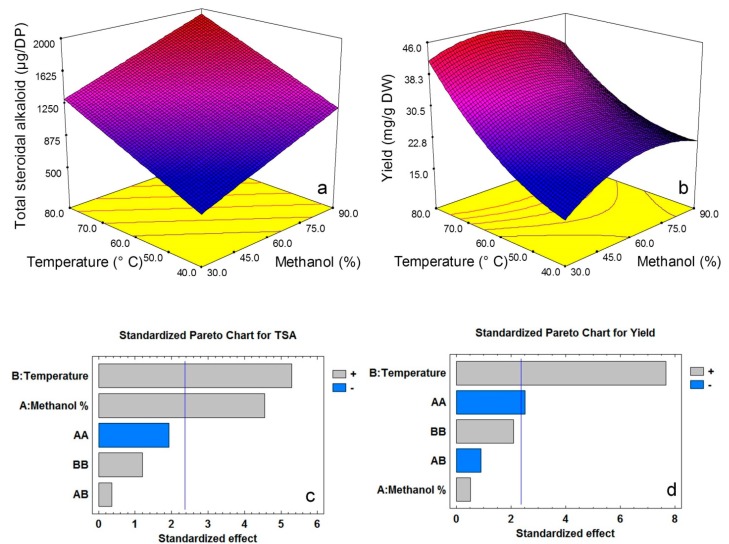
Response surface plots and standardised pareto chart of the effect of temperature and methanol concentration on extraction yield (**a**,**c**) and total steroidal alkaloids (**b**,**d**) content in potato peel pressurized liquid extracts. Bars in pareto chart exceeding the vertical line on the graph indicate that the corresponding factor terms are significant (*p* < 0.05).

The extraction of total steroidal alkaloids was affected by temperature and methanol concentration at linear scale (Figure 4a,c). There was no quadratic effect of the factors on extraction of total steroidal alkaloids. However, extraction yield had significant (*p* < 0.05) quadratic effect for methanol concentration although temperature was the dominant factor to enhance extraction yield (Figure 4b,d). Solvent mixture plays an important role to improve extraction efficiency. In a dual mixture such as methanol/water, named as methanol concentration in the current study, one solvent might facilitate solubility of the target analyte while the other solvent might accelerate analyte desorption.

### 2.2. Model Fitting

Prior to garnering any information from the developed model it is important to check the significance of the models. The high F-values (Table 2) of the models as obtained from analysis of variance (ANOVA) indicated that the models were significant. The tests of significance of the models (*p* ≤ 0.0005) were also in agreement with the F-values. Moreover, models ‘lack of fit’ data were not significant.

The analysis of variance showed that the R-squared statistic of all the parameters was in the range of 0.780 to 0.846 indicating high representation of the variability of the parameters by the models (Table 2). The predicted values obtained by the quadratic polynomial equations showed strong correlation with actual experimental values with Pearson’s correlation coefficients (r) from 0.883 to 0.919.

**Table 2:** Analysis of the variance of the models and predictive second order polynomial equations for different glycoalkaloids in PLE of potato peel.

Parameters	R^2^	CV	p	Lack of Fit	F-value	Second Order Polynomial Equation
α-solanine	0.784	13.55	0.0005	0.613	18.55	Y = −172.904 + 3.53787 × X_1_ + 5.70597 × X_2_
α-chaconine	0.780	12.05	0.0005	0.322	17.70	Y = −166.793 + 4.45751 × X_1_ + 8.06025 × X_2_
Solanidine	0.783	12.74	0.0005	0.536	18.02	Y = −91.2571 + 1.98821 × X_1_ + 3.61989 × X_2_
Demissidine	0.795	12.34	0.0004	0.503	19.42	Y = −18.501 + 0.403972 × X_1_ + 0.720972 × X_2_
Total alkaloids	0.790	12.36	0.0004	0.478	18.79	Y = −449.032 + 10.3792 × X_1_ + 18.1071 × X_2_
Yield	0.846	10.66	0.0005	0.177	16.46	Y = −24.3625 + 0.794455 × X_1_ + 0.525292 × X_2_ − 0.0064205 × X_1_^2^

### 2.3. Optimisation and Model Validation

The response surface methodology (RSM) guided optimisation demonstrated that the optimum treatment for maximising the extraction of steroidal alkaloids from potato peel was 79.6 °C and 89.3% methanol. As the PLE system does not allow a decimal point in choosing the parameters, the validation experiment was carried out at 80 °C using 89% methanol. Using these extraction parameters a total steroidal alkaloids content of 1919 µg/g dried potato peel (597, 873, 374 and 75 µg/g dried potato peel for α-solanine, α-chaconine, solanidine and demissidine respectively) were produced in the PLE (Table 3). Whilst for the conventional SLE yield observed was 981 µg/g dried potato peel (247, 474, 224 and 36 µg/g dried potato peel extract for α-solanine, α-chaconine, solanidine and demissidine respectively) (Table 3).

**Table 3:** Predicted and experimental values of the parameters tested at optimal PLE condition in comparison to the conventional solid/liquid extraction values and average mean deviation between predicted and experimental values of optimal PLE (80 °C and 89% methanol).

Parameter	Predicted Values (79.6 °C, 89.3% MeOH)	Experimental Values (80 °C, 89% MeOH)	(E%)	Solid/Liquid Extraction Values
α-Solanine (µg/g DW)	579.9	597.4 ± 6.48	3.01	247.3 ± 7.54
α-Chaconine (µg/g DW)	851.0	873.0 ± 15.26	2.58	474.0 ± 11.23
Solanidine (µg/g DW)	365.0	374.0 ± 9.87	2.46	224.2 ± 8.57
Demissidine (µg/g DW)	73.0	75.0 ± 2.56	2.73	36.0 ± 5.46
Total steroidal alkaloid (mg/g DW)	1.87	1.95 ± 0.03	4.27	0.98 ± 0.02
Yield (mg/g DW)	39.2	37.8 ± 0.56	3.57	30.12 ± 0.78

Since there is no report of optimisation of PLE on steroidal alkaloids this far, data from the current study could not be compared with literature data. However, Wijngaard *et al.* [20] identified the optimal PLE of antioxidant phenolic compounds from potato peels as 70% ethanol at 125 °C. This extraction condition was also used to isolate total steroidal alkaloids, value of which was not significantly higher than those of SLE. Since polyphenols, particularly phenolic acids, which are the predominant polyphenols in potato peels and steroidal alkaloids are different in their chemical nature, polarity and solubility, that the optimal extraction condition for polyphenols could not be considered as the optimal condition for steroidal alkaloids. Hence determination of the optimal key extraction conditions (% organic solvent and temperature) for steroidal alkaloids was necessary. The optimal PLE conditions in the current study are very close to the highest parameters used in this RSM model. Therefore, extending the levels of the factors *i.e.* 10% increase in methanol concentration and temperature rise beyond 80 °C might increase the steroidal alkaloids contents of the extracts, but this will further compromise the criteria of using minimal organic solvent and energy for an environmentally friendly extraction method [25]. The predicted values from the developed second order quadratic polynomial equation were in close agreement with the experimental values with low average deviation of means (E%) (Table 3); therefore, the predictive performance of the established model may be considered acceptable. The extraction yields of the optimal PLE extracts were significantly (*p* < 0.05) higher than the solid–liquid extracts.

## 3. Experimental Section

### 3.1. Samples and Reagents

Potato peel slurry was kindly provided by Largo Foods Limited (Meath, Ireland). The steroidal alkaloids such as α-solanine, α-chaconine, solanidine and demissidine were purchased from Extrasynthese (Genay Cedex, France). HPLC grade solvents methanol, water, formic acid and acetonitrile were purchased from Sigma-Aldrich (Wicklow, Ireland). Reagent grade tetrahydrofuran, methanol, ethylacetate, chloroform, acetonitrile, acetone, isopropanol, ethanol, dichloromethane and acetic acid were purchased from Sigma-Aldrich (Wicklow, Ireland).

### 3.2. Drying of the Peel Slurry

The peel slurry was pressed by hand to remove excessive water. Then the peels were spread on aluminum trays and allowed to be frozen at −40 °C before putting them into the freeze drier. Freeze-drying was carried out in a Frozen in Time Limited (York, UK) freeze-drier at a temperature of −54 °C and a pressure of 0.064 mbar for 72 h. All the dried samples were immediately powdered, vacuum packed and kept in −20 °C for extraction within two weeks. The film (75 μm thickness) of the vacuum pack pouches (Allfo Vakuumverpackungen Hans Bresele KG, Germany) were composed of a mixture of polyamide and polyethylene with oxygen and carbon dioxide permeability rate of 60 cm^3^/m^2^/24 h/atom and 180 cm^3^/m^2^/24 h/atom respectively (23 °C, 75% RH). The water vapour permeability of the film was 2.7 g/m^2^/24 h at 23 °C and 85% RH.

### 3.3. Pressurized Liquid Extraction (PLE)

PLE was performed on a Dionex ASE 200 (Dionex Corp., Sunnyvale, CA, USA) system. Dried and powdered potato peels (1.0 g) were placed in between two layers of diatomaceous earth in a 22 mL Dionex (ASE 200) stainless-steel cell. The cells were equipped with a stainless steel frit and a cellulose filter (Dionex Corp.) at the bottom to avoid the collection of suspended particles in the collection vial. A dispersing agent (diatomaceous earth) was used to reduce the solvent volume used for the extraction. The extraction cell was arranged in the cell tray and was extracted using conditions specified using RSM guided experimental design (40–80 °C and 30%–90% methanol). The automated PLE cycle was as follows: the cell containing the sample was pre-filled with the extraction solvent (previously degassed with N_2_), pressurized (1500 psi), and then heated for 5 min followed by a static period of 5 min. The sample was extracted with methanol at the concentration and temperature as specified in the experimental design during this 5 min. Then the cell was rinsed with fresh extraction solvent (60% of the extraction cell volume) and purged with a flow of nitrogen (150 psi during 90 s). Extracts (34 mL) were collected in 60 mL glass vials. The extracts were stored at −20 °C in darkness until required for steroidal alkaloids analysis. The experiment was performed in two batches which included three replications for each sample. Prior to UPLC-MS/MS analysis the extract was filtered through a 0.45 µm PTFE filters (Millipore, Billerica, MA, USA).

### 3.4. Conventional Solid/Liquid Extraction

Solid–liquid extractions were carried out according to the method of Shan *et al.* [11] with slight modifications. Briefly, dried and ground samples (1.0 g) were homogenized for 1 min at 24,000 rpm using an Ultra-Turrax T-25 Tissue homogenizer (Janke & Kunkel, IKA^®^-Labortechnik, Saufen, Germany) in 25 mL of 90% methanol in water at room temperature (~23 °C). The homogenized sample suspension was shaken for 60 min with a V400 Multitude Vortexer (Alpha laboratories, North York, ON, Canada) at 1500 rpm at room temperature. The sample suspension was then spun for 15 min at 2000 *g* (MSE Mistral 3000i, Sanyo Gallenkamp, Leicestershire, UK) and immediately filtered through 0.22 µm polytetrafluoethylene (PTFE) filters. The extracts were kept at −20 °C until subsequent analysis. The experiment was performed in two batches which included three replications for each sample. Extraction yield data was calculated as the amount (mg) of dried extract obtained from 1 g of dried potato peel.

### 3.5. Identification and Quantification of Steroidal Alkaloids in Potato Peel by Ultra-Performance Liquid Chromatography Coupled with Tandem Mass Spectrometry

Steroidal alkaloids were analysed using Waters Acquity (Waters Corporation, MA, USA) ultra-performance liquid chromatography coupled with tandem mass spectrometry (UPLC-MS/MS) as described previously [26]. The compounds were separated on a Waters Acquity BEH C18 column (50 × 2.1 mm, particle size 1.7 µm) using 0.5% formic acid in water (Solvent A) and 0.5% formic acid in acetonitrile (solvent B) . The following gradient program was carried out: 10% B to 21% B in 0.5 min, 21% B until 4.5 min, 40% B in 6 min, 40% B until 7 min, 90% B in 7.5 min, 90% B until 8.5 min, 10% B in 9 min and 10% B until 10 min at a flow rate of 0.5 mL/min. The injection volume for all the samples was 5 μL. All the standards in the concentration range from 0.1 to 1 µg/mL for quantification purposes were dissolved in methanol. Samples for UPLC-MS/MS analysis were prepared in (methanol) following the extraction described in the above Section 3.4. The UPLC-MS/MS experiments were performed in the positive ionisation mode. The ionization source conditions were as follows: capillary voltage 3 kV, Cone voltage 30 V, extractor voltage 3 V, source temperature 120 °C, desolvation temperature 350 °C, desolvation gas flow 800 L/h, cone gas flow 50 L/h, and collision gas flow 0.10 mL/min. The multiple reaction monitoring (MRM) transition spectral data were acquired using the Waters MassLynx V4.1^TM^ software while the quantifications of the data were carried out using the Waters TargetLynx^TM^ software. The data (area under the curve) acquired for respective masses were used to obtain the standard curve (R^2^ = 0.991).

### 3.6. Statistical Analysis

Optimal PLE extraction conditions were determined by RSM which was performed using the Design Expert Version 7.1.3 software (Stat-Ease, Inc., Minneapolis, MN, USA). A central composite design (CCD) was used to investigate the effects of two independent variables (solvent concentration and extraction temperature) on the dependent variables (content of α-solanine, α-chaconine, solanidine, demissidine, extraction yield and total steroidal alkaloid content). The data obtained from the CCD design was fitted with a second order polynomial equation (Equation (1)). The equation was as follows:

(1) $$Y=\beta_{0}+\sum_{i-1}^{2}\beta_{i}X_{i}+\sum_{i-1}^{2}\beta_{ii}X_{i}^{2}+\sum_{i}\sum_{j=i+1}\beta_{ij}X_{i}X_{j}$$

where *Y* is the predicted response; *β*_0_ is a constant; *β_i_* is the linear coefficient; *β_ii_* is the quadratic coefficient, *β_ij_* is the interaction coefficient; and *Xi* and *Xj* are independent variables. The adequacy of the model was determined by evaluating the lack of fit, coefficient of regression (R^2^) and the Fisher test value (F-value) obtained from the analysis of variance (ANOVA). Statistical significance of the model and model variables was determined at the 5% probability level (*p* < 0.05). The software uses the quadratic model equation shown above to build response surfaces. Three-dimensional response surface plots were generated by keeping one response variable at its optimal level and plotting that against two factors (independent variables). The complete design consisted of 13 experimental points including five replications of the central point.

### 3.7. Model Validation

The predictive performance of the developed models describing the combined effect of amplitude (X_1_), temperature (X_2_) and time (X_3_) on independent variables (α-solanine, α-chaconine, solanidine and demissidine) of potato peel were validated with optimal extraction conditions as predicted by the design.

The criterion used to characterize the fitting efficiency of the data to the model was the multiple correlation coefficients (R^2^) and their average mean deviation (E, Equation (2)).

(2) $$E\left(\%\right)=\frac{1}{n_{e}}\sum_{i=1}^{n}\left\|\frac{V_{E}-V_{P}}{V_{E}}\right\|\times 100$$

where, *n*_e_ is the number of experimental data, *V*_E_ is the experimental value and *V*_P_ is the predicted value.

## 4. Conclusions

Steroidal alkaloids content of the PLE extracts of the potato peel increased significantly with the increase of temperature. PLE temperature of 80 °C showed the best glycoalkaloid yield. Methanol concentration also showed significant effect and recorded the highest alkaloid content at 89% aqueous methanol. Results of the model validation showed that the developed model had acceptable predictive performance as assessed by mathematical and graphical model performance indices. PLE extracts optimized at 80 °C and 89% methanol for steroidal alkaloids showed significantly (*p* < 0.05) higher yield values than the conventional solid/liquid extracts. Therefore optimised PLE could be used as a high throughput extraction system for extracting steroidal alkaloids.
